# Missing links exploring transitions

**DOI:** 10.1007/s00709-023-01902-1

**Published:** 2023-10-17

**Authors:** Peter Nick

**Affiliations:** Joseph Gottlieb Kölreuter Institute für Plant Sciences, Karlsruhe Institute of Technology, Karlsruhe, Germany

Life is change, but the results of this change often appear as discrete states, not revealing the transitions between them. These can often only be inferred, not observed. This limitation holds true for the compartments of a cell that appear as organelles of defined and constant shape, but also for the development of individual organisms, as well as for the transformation of species. The missing of the links is, therefore, a central problem of any theory that is considering transformations. Darwin’s friend and mentor, the geologist Charles Lyell, introduced the concept of missing links to explain gaps in the geological record, but soon adopted it to problems of biological evolution, for instance in the transition between apes and humans (Lyell 1863). The discovery of the famous *Archeopteryx* in Solnhofen 1861 was generally seen as a breakthrough for Darwin’s theory of continuous change, because it showed both ancestral reptile and modern bird traits and, thus, almost perfectly illustrated the concept of a missing link. As spectacular as such finds may be, they can easily trap our conceptual thinking, because we are tempted to align such transitional forms along a linear and steady path, leading from one state to the other. A closer look often uncovers other transitional forms that are similar, but combine ancestral and prospective traits in a slightly different manner and, therefore, are usually not easily integrated into a linear path. It rather seems that transitions are more appropriately conceived as a reticulate zone, where many dead ends obscure the paths bridging the states, and where each transition defined as “the” missing link represents a different and individual manifestation of old and new. The more fundamental the transition, the more likely we will never find “the” missing link, but instead an entire cloud of them that represent different variations of the same theme, but are obviously occurring as parallel and not as consecutive states.

The contribution by Holzinger et al. (2023) has to be read in the context of a crucial step in evolution—the transition to a terrestrial lifestyle. The methodological advances in genomics over the last decade have allowed us to leave the narrow realm of the canonical model organisms and address more remote life forms. It has become clear now that this transition was not a single event, but that different algal taxa have, to different degrees, managed to cope with the harsh conditions of terrestrial habitats (for review see de Vries and Archibald 2018). Interestingly, the step on land was not launched from the sea, but from freshwater. The progress in phylogenomics, as insightful as it has been, remains incomplete, if not accompanied by investigations on the cellular physiology of those organisms that dwell at the transition from water to land. This is exactly the topic addressed in this study on *Trentepohlia*, a genus belonging to the Ulvophyceae, a group of green algae. In contrast to its marine counterparts, such as the Sea Lettuce (*Ulva lactuca*), *Trentepohlia* has conquered very harsh terrestrial habitats, leading to the question, by what mechanisms it is able to do so. The authors have collected three taxa, from rock and tree bark, and classified them with fluorescent dyes for components of the cell wall and the membrane system. Since their physiology seemed untouched by their challenging environment, they were exposed to severe desiccation stress followed by re-hydration, monitoring parameters of photosynthetic activity. All three species could recover photosynthesis, two of them even to a level as if there had been no stress at all. All species are conspicuously coloured, due to an extremely high content of carotenoids. The impressive stress resilience was linked to a high content of sugar alcohols, such as mannitol, arabitol, or erythritol. This metabolic trait correlated with the ability for photosynthetic recovery, congruent with previous studies, where these sugar alcohols were shown to act as compatible solutes in microalgae (Gustavs et al. 2010). The recovery after stress was not caused by the rapid proliferation of few surviving cells, but by their persistently high viability even during the osmotic challenge. This conclusion can be drawn from stains with the fluorochrome Auramine O, a vitality marker suited to address the stress tolerance of green algae in general (Trumhová et al. 2019). Interestingly, the three species differ in the degree to which they have conquered the transition to the harsh conditions of their new habitat, boosting protective metabolic activities, such as carotenoid synthesis and the accumulation of sugar alcohols. These metabolic activities are present in all green algae. In other words, nature did not need to invent a novel breakthrough innovation to expand to solid land but just drew on pre-existing traits by amplifying and de-regulating them. A second conclusion might be that *conquering* a new habitat probably started very modestly as *enduring* a new habitat. This step was successfully achieved by several algal groups, even from those taxa that were not part of the lineage leading to the “real” land plants.

An equally important transition was the invention of a chorda that later became the organising body axis of the vertebrates. This transition was linked with a fundamental innovation within the intermediate filaments, a cytoskeletal element that, unlike microtubules and actin filaments, is fairly stable and able to buffer considerable tension forces. This transition is scrutinised in the contribution to the current issue by Karabinos (2023). A specific subset of these proteins, the nuclear lamins, are responsible for the shape and architecture of the metazoan nucleus and are thought to have evolved very early in eukaryotic evolution. They are endowed with a specific CaaX motif recruiting an isoprenyl anchor, such that a network can form that sustains the form and architecture of the nucleus in many eukaryotes (Peter and Stick 2023). In addition to these nuclear lamins, there exist two types of cytoplasmic filaments. One of them is long and shares a long, coiled domain with the nuclear lamin – this type is found in the Protostomia. The second type is short, mainly due to a large deletion within this coiled domain. This short type is found in the Deuterostomia. Two concurrent hypotheses try to explain the relationship between the three types of intermediate filament proteins. A straightforward idea suggests that both cytoplasmic protein types are derived from a nuclear lamin ancestor. A second, more complex, hypothesis assumed that the long cytoplasmic intermediate filament proteins had to be strictly confined to prevent interaction with nuclear lamins. In fact, in the Protostomia, they are only encoded by very few genes, and also expressed under tight control. The deletion in the coiled domain removed this constraint because it prevented this interaction, such that cytoplasmic intermediate filaments could be expanded considerably, which was one of the conditions enabling the chorda. The author investigates the intermediate filament genes in different species of *Branchiostoma*, the prime model for the initiation of chordate evolution. He finds that these organisms harbour both, the long protostomic and the short deuterostomic type of proteins. However, the long protostomic protein, lacking the deletion in the coiled domain, is found only in Branchiostoma, not in any other chordate or vertebrate. So-called type III and IV intermediate filament proteins that are important in vertebrates are missing completely. This pattern lends support to the second model, where the deletion in the coiled domain safeguarded the cytoplasmic proteins against interaction with nuclear lamins and enabled their expansion.

As different as the two case studies are, they can teach us some general lessons about transitions in evolution. Such transitions are rarely sharp boundaries, but rather appear as fuzzy searching zones, where “old” and “new” are combined in different ways, often in an incomplete manner. So, the Ulvaceae have acquired some of the cellular mechanisms providing endurance of a terrestrial lifestyle, while lacking others found in Bryophytes or primordial cormophytes. To see them as “evolutionary dead-ends” would be inappropriate, however. They cope with the conditions of their harsh environment perfectly. The idea of a “dead-end” is only brought about by our teleological thinking, where “primitive” forms are seen as transitions for something more advanced. In the same way, the mechanism to downregulate the intermediate-filament proteins without the large coil deletion is not “more primitive” than the “advanced” deletion-bearing version of deuterostomates. The seeming advance comes only from our way of interpreting it as “pre-adaptation” (ex-aption in sensu Gould and Vrba 1982) for the later evolutionary stages that were enabled by this deleted version, such as the genesis of chorda as an organisational principle of the vertebrates.

A second lesson is the type of change that leads to “innovation” (defined as such only *a posteriori*). These changes seem to be often of a de-regulative nature. For instance, a de-regulated accumulation of carotenoids, in concert with a de-regulated accumulation of sugar alcohols helps *Trentepohlia* to cope with drought and light stress. The de-regulated expression of cytoplasmic intermediate-filament proteins in the early chordates (possibly due to the deletion in the coiled domain because this way evades interaction with nuclear lamins) enables the development of a chorda. Such de-regulations are easy to get in evolution because they are caused by loss-of-function of negative regulators. To reach a loss-of-function is far easier than to assemble a new function. Thus, it might be the loss of control that allows for evolutionary innovation, “unleashing the creativity of Nature”.
